# Antibodies to cyclic citrullinated protein and erythrocyte sedimentation rate predict hand bone loss in patients with rheumatoid arthritis of short duration: a longitudinal study

**DOI:** 10.1186/ar2749

**Published:** 2009-07-01

**Authors:** Pernille Bøyesen, Mari Hoff, Sigrid Ødegård, Glenn Haugeberg, Silje W Syversen, Per I Gaarder, Cecilie Okkenhaug, Tore K Kvien

**Affiliations:** 1 Department Rheumatology, Diakonhjemmet Hospital, Diakonveien 12, N-0370 Oslo, Norway; 2 Department of Rheumatology, St Olav's Hospital, University Hospital of Trondheim, Olav Kyrres gt 17, N-7006 Trondheim, Norway; 3 Department of Rheumatology, Sørlandet Hospital, Service box 416, N-4604 Kristiansand S., Norway; 4 Department of Immunology and Transfusion Medicine, University Hospital Ullevål, Kirkeveien 166, N-0459 Oslo, Norway; 5 Department of Medical Biochemistry, Diakonhjemmet Hospital, Diakonveien 12, N-0370 Oslo, Norway

## Abstract

**Introduction:**

Radiographic progression in rheumatoid arthritis (RA) has in several studies been shown to be predicted by serological markers widely used in daily clinical practice. The objective of this longitudinal study was to examine if these serological markers also predict hand bone mineral density (BMD) loss in patients with RA of short disease duration.

**Methods:**

163 patients with RA of short disease duration (2.4 years) were included and followed longitudinally. Antibodies to cyclic citrullinated protein (anti-CCP), rheumatoid factor (RF), erythrocyte sedimentation rate (ESR), and C-reactive protein (CRP) were analysed from baseline blood-samples. Hand BMD was measured by digital X-ray radiogrammetry (DXR) based on hand and wrist radiographs obtained at baseline and 1, 2 and 5-year follow-up.

**Results:**

During the study period, DXR-BMD decreased by median (inter quartile range) 1.7% (4.1 to 0.4), 2.8% (5.3 to 0.9) and 5.6% (11.7 to 2.3) after 1, 2 and 5 years, respectively. Elevated baseline anti-CCP, RF, ESR and CRP levels were in univariate linear regression analyses consistently associated with DXR-BMD change at all time-points. Anti-CCP and ESR were independently associated with hand DXR-BMD in multivariate linear regression analyses. Elevated anti-CCP levels were consistent and independent predictors of loss in cortical hand bone during the study period, with the odds ratios (95% confidence interval) 2.2 (1.0 to 4.5), 2.6 (1.1 to 6.2) and 4.9 (1.4 to 16.7) for the 1, 2, and 5-year follow-up periods, respectively.

**Conclusions:**

Anti-CCP and ESR were found to be independent predictors of early localised BMD loss. This finding adds to the understanding of anti-CCP and ESR as important predictors of bone involvement in RA.

## Introduction

Rheumatoid arthritis (RA) is a chronic inflammatory disease characterised by synovitis and bone destruction. The inflammation in RA causes a shift in the bone metabolism towards increased osteoclast-mediated bone turn-over [1,2]. This dysregulation causes reduced bone mass, which is known to be an early feature in RA patients, visualised as juxta-articular bone demineralisation on radiographs [3]. Quantification of this localised bone loss has been proposed as an outcome measure in early RA [4]. Measurements of localised bone involvement in RA can be performed by digital X-ray radiogrammetry (DXR), which gives an estimate of cortical hand bone mineral density (BMD) [5,6].

Early intervention with disease-modifying antirheumatic drugs (DMARDs), which inhibit joint damage, is accepted as a cornerstone in the treatment strategy of RA [7,8]. Further, the disease course of RA is heterogeneous and about one-third of RA patients do not experience joint damage [9,10]. Thus, the identification of patients prone to bone involvement is important at an early stage of the disease in order to individually tailor the RA treatment and optimise disease outcome [1]. DXR has been shown to measure bone loss in early arthritides and RA [11]. As a measurement of early bone destruction in RA, DXR-BMD has also been shown to predict subsequent radiographic damage [12]. Previous studies have shown that serological biomarkers can predict radiographic damage, a late measure of bone involvement in RA [13].

The objective of this study was to examine if serological markers widely used in daily clinical practice also can predict early involvement of bone measured by DXR in a longitudinal study of patients with RA of short disease duration.

## Materials and methods

### Patients

As part of the EURIDISS (European Research on Incapacitating Disease and Social Support) study, a Norwegian arm of the cohort was followed longitudinally. At inclusion in 1992, 238 patients aged from 20 to 70 years, with a clinical diagnosis of RA and disease duration of less than four years were included [14]. The patients were assessed at baseline with blood samples, medical history and health assessment questionnaire (HAQ). Conventional, bilateral hand and wrist radiographs were taken at baseline and one, two and five-year follow-up. This article focuses on 163 patients who had radiographs taken at baseline and after one, two or five years follow-up. Of the 163 patients in this study, 128 had X-rays at all four time points, 29 at three time points and six patients at two time points. The patients with and without hand X-rays had similar baseline characteristics (Table 1). Treatment was given according to clinical practice. The percentages of patients who were treated with DMARDs/prednisolone at baseline, one, two and five years were 53.8/26.3, 46.9/28.1, 50.6/29.4 and 54.9/37.5, respectively. The included patients gave informed consent and the study was evaluated and approved by the regional ethics committee.

**Table 1:** Baseline demographics, treatment and levels of serological biomarkers

	Included patients n = 163	Excluded patients n = 75	*P *value
**Demographic variables**			
Female	75.0	70.5	0.53
Age (years)	53.0 (43.0 to 62.3)	57.0 (43.5 to 64.5)	0.23
Disease duration (years)	2.4 (1.2 to 3.2)	2.5 (1.2 to 3.1)	0.95
HAQ	0.9 (0.4 to 1.4)	1.0 (0.4 to 1.4)	0.60
**Treatment**			
DMARD use	53.8	48.7	0.49
Prednisolone use	26.3	29.5	0.64
**Serological biomarkers**			
ESR (mm/h)	20.0 (10.0 to 38.0)	24.0 (10.5 to 35.5)	0.65
ESR > 20 mm/h	48.5	52.0	0.58
CRP (mg/l)	6.0 (0.0 to 15.0)	7.0 (2.5 to 14.5)	0.36
CRP > 10 mg/l	28.8	26.0	0.66
IgA RF (U/ml)	13.0 (4.0 to 41.3)	17.5 (1.8 to 75.0)	0.56
IgA RF positive	30.2	33.3	0.87
IgM RF (U/ml)	21.0 (5.0 to 105.0)	26.5 (2.0 to 131.0)	0.66
IgM RF positive	41.7	66.7	0.40
Anti-CCP (U/ml)	67.0 (4.4 to 243.0)	56.0 (3.5 to 251.0)	0.82
Anti-CCP positive	60.4	66.7	0.76

The values are given as median (inter quartile range) for continuous variables, percentage for counts.

Anti-CCP = antibodies to cyclic citrullinated peptide; CRP = C-reactive protein; DMARD = disease-modifying antirheumatic drugs; ESR = erythrocyte sedimentation rate; HAQ = health assessment questionnaire; Ig = immunoglobulin; RF = rheumatoid arthritis.

### Laboratory analyses

Erythrocyte sedimentation rate (ESR) was measured by the Westergren method, ranging from 0 to 140 mm/h. C-reactive protein (CRP) was measured by phyCardioPhase hs CRP nephelometry (Dade Behring, Deerfield, Illinois, USA) with a lowest detectable limit of 0.15 mg/l [9]. Antibodies to cyclic citrullinated protein (anti-CCP) was analysed by a second generation ELISA (INOVA Diagnostics Inc, San Diego, CA, USA) with a range from 0 to 251 U/ml. Values above 25 U/ml were considered positive. Immunoglobulin (Ig) A and IgM rheumatoid factor (RF) were measured by in-house ELISA technique, ranging from 2 to 300 U/ml and with a positive cut-off at 25 U/ml [9]. The laboratory analyses used in this study were performed on baseline samples and the measures of CRP, anti-CCP and RF were performed in frozen sera.

### Bone mineral density measurement of the hands

BMD was measured by DXR (Pronosco X-posure 2.0, Sectra, Linköping, Sweden) based on conventional hand radiographs from baseline, one, two and five-year follow-up visits. DXR is a computer version of the traditional radiogrammetry technique and the method has previously been described in detail [6]. The DXR software automatically recognises the regions of interest (metacarps two to four) and measures the cortical thickness, bone width, and bone porosity 118 times per cm. The precision of the DXR-BMD measurements was calculated based on duplicate hand radiographs from 28 healthy individuals with repositioning of the hand between each measure. The coefficient of variation was found to be 0.28%, and the least significant change (LSC) was 0.79% [12]. Mean values of both hands were applied to avoid bias regarding dominant and non-dominant hand and to achieve better precision [15].

### Statistical analyses

The analyses were performed using SPSS 14 statistics package (SPSS, Chicago, IL, USA). The baseline characteristics had a skewed distribution and were analysed using non-parametric methods. Independent groups were compared using Mann-Whitney U test for continuous variables and chi-squared tests for dichotomous variables. DXR-BMD change was calculated as the percentage difference between the follow-up value and the baseline value. The individual zero to one, one to two and two to five years changes in DXR-BMD were illustrated by cumulative probability plots. DXR-BMD change was also depicted in probability plots stratified for anti-CCP more than 25 U/ml and 25 U/ml or less [16]. The distributions of the soluble biomarkers were skewed (independent variables), and were therefore for further analyses dichotomised according to the clinical cut-offs with elevated levels as follows: ESR above 20 mm/h, CRP above 10 mg/l, IgA RF above 25 U/ml, IgM RF above 25 U/ml and anti-CCP above 25 U/ml.

The associations between the change in DXR-BMD and the baseline, dichotomised soluble biomarkers were explored by linear regression analyses. First, univariate linear regression analyses were performed with one, two and five-year change in BMD as dependent variables and the dichotomised soluble biomarkers as independent variables. Further, the independent variables with a *P *≤ 0.25 were included in multivariate linear analyses. The final multivariate models with only statistically significant variables were obtained by stepwise exclusion of the least significant variable from the models and corrected for age and sex.

Prediction of loss in cortical hand bone was further explored by logistic regression analyses. Loss in cortical hand bone was defined as a negative change in DXR-BMD exceeding the LSC. First, univariate logistic regression analyses were performed with one, two and five-year cortical hand bone loss as the dependent variable and the baseline, dichotomised serological biomarkers as independent variables. Secondly, the independent variables with *P *≤ 0.25 were included in multivariate logistic regression analyses. By stepwise exclusion of the least significant covariate, final models with only statistically significant variables were obtained and corrected for age and sex.

All tests were two-sided and *P *≤ 0.05 were considered to be statistically significant. Standard diagnostic tests of model assumptions and residuals were routinely performed. Residuals exceeding three standard deviations were checked for data errors.

## Results

### Baseline demographics and soluble biomarkers

Baseline demographics for included and excluded patients were similar and are summarised in Table 1.

### Bone mineral density

**Figure 1:**
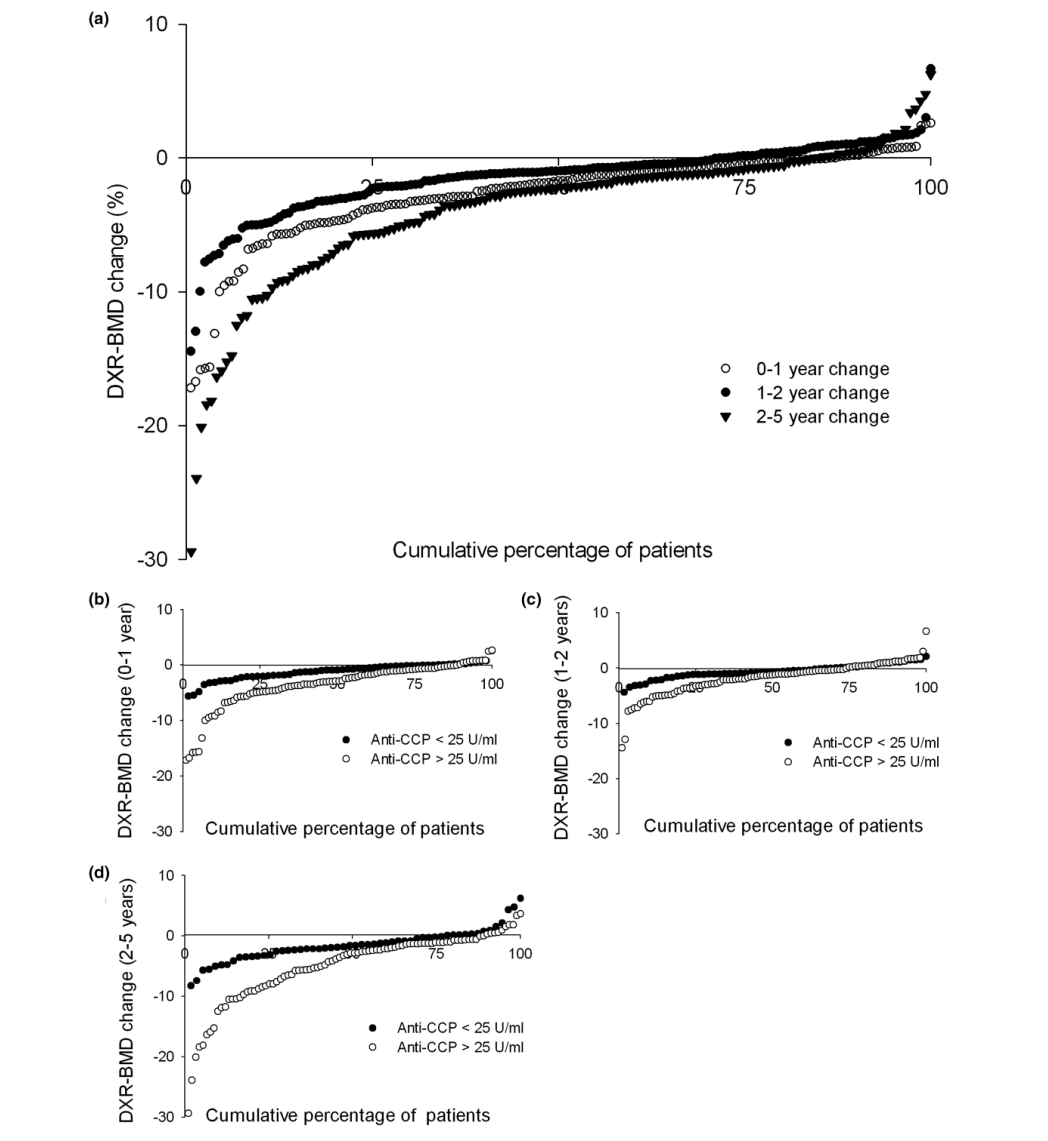
Cumulative probability plots presenting the individual patients' change in DXR-BMD. **(a) **Change in digital X-ray radiogrammetry bone mineral density (DXR-BMD) of the entire study group (0 to 1 years, 1 to 2 years, 2 to 5 years). **(b-d) **Change in DXR-BMD stratified according to antibody to cyclic citrullinated proteins (anti-CCP) positive/negative patients (empty circle: anti-CCP > 25 U/ml, filled circle: anti-CCP ≤ 25 U/ml) for 0 to 1, 1 to 2 and 2 to 5 year change, respectively.

DXR-BMD measurements from each examination time point and DXR-BMD change are presented in Table 2. DXR-BMD decreased significantly between all time points during the follow-up period (*P *< 0.05). Median (inter quartile range) DXR-BMD loss between one and two years, and two and five years were -1.46% (-1.88 to -1.04) and -3.81% (-4.68 to -2.95; Figure 1a). Younger women (≤ 50 years) had a trend towards larger DXR-BMD loss than older women (> 50 years). Median one, two and five-year DXR-BMD change in younger women were -2.32%, -3.39% and -7.45% and in older women -1.15%, -1.73% and -3.88%, respectively. However, this trend was the same for the men included in the study (data not shown). DMARD/prednisolone-treated patients had significantly larger one, two and five-year DXR-BMD percentage loss than patients not treated with DMARD/prednisolone; -2.0/-2.2 vs. -1.1/-1.3, -3.4/-4.1 vs. -1.5/-1.5 and -7.7/-7.8 vs. -3.3/-3.9, respectively. A loss in cortical hand bone exceeding the LSC was observed in 66.7%, 77.3% and 89.1% of the patients at one, two and five-year follow-up, respectively (Table 2).

**Table 2:** DXR-BMD measurements

	Baseline (n = 163)	One-year follow-up (n = 156)	Two-year follow-up (n = 154)	Five-year follow-up (n = 138)
DXR-BMD (g/cm^2^)	0.560 (0.491 to 0.608)	0.545 (0.470 to 0.590)	0.528 (0.458 to 0.586)	0.508 (0.427 to 0.572)
DXR-BMD change (%) †		-1.72 (-4.07 to -0.36)	-2.80 (-5.29 to -0.86)	-5.58 (-11.72 to -2.31)
Patients with loss in cortical hand bone (%) ‡		66.7	77.3	89.1

Median (inter quartile range) for continuous variables, percentage for counts. Number of patients presented in brackets. DXR-BMD = digital X-ray radiogrammetry bone mineral density. † DXR-BMD change from baseline ‡ Decrease in DXR-BMD exceeding the least significant change (least significant change = -0.79%).

### Associations between baseline serological biomarkers and change in DXR-BMD

A trend towards larger loss in DXR-BMD in RA patients with elevated levels of anti-CCP compared with patients with low levels was observed in cumulative probability plots (Figure 1b to 1d). Similar trends were seen elevated vs. low levels of ESR, CRP and RF (data not shown).

Possible associations between DXR-BMD and the serological biomarkers were explored in linear regression analyses with DXR-BMD loss as the dependent variable. Elevated baseline levels of anti-CCP, RF, ESR and CRP were associated with an increased one, two and five year DXR-BMD loss in univariate linear regression analyses (Table 3). Age, HAQ, and DMARD and prednisolone treatment were also associated with DXR-BMD loss at all time points (Table 3).

**Table 3:** Univariate associations between change in DXR-BMD, baseline characteristics and baseline serological biomarkers (univariate linear regression analyses)

	One-year change in DXR-BMD (%)		Two-year change in DXR-BMD (%)		Five-year change in DXR-BMD (%)	
			
	B (95% CI)	*P *value	B (95% CI)	*P *value	B (95% CI)	*P *value
Age (years)	-0.1 (-0.1 to 0.0)	0.03	-0.1 (-0.2 to 0.0)	0.002	-0.2 (-0.3 to 0.0)	0.004
Sex (female)	-0.1 (-1.4 to 1.2)	0.90	0.6 (-1.3 to 2.4	0.54	0.0 (-3.0 to 3.1)	0.98
Disease duration (years)	0.1 (-0.4 to 0.6)	0.74	0.2 (-0.5 to 0.9)	0.66	0.8 (-0.4 to 1.9)	0.18
HAQ	-1.4 (-2.2 to -0.5)	0.003	-1.9 (-3.1 to -0.7)	0.003	-2.3 (-4.5 to -0.2)	0.03
ESR (> 20 mm/h)	-2.9 (-4.0 to -1.9)	< 0.001	-4.2 (-5.7 to -2.8)	< 0.001	-6.6 (-9.0 to -4.1)	> 0.001
CRP (> 10 mg/l)	-1.6 (-2.8 to -0.3)	0.01	-3.4 (-5.1 to -1.7)	< 0.001	-3.9 (-6.8 to -1.0)	0.008
Anti-CCP (> 25 U/ml)	-2.3 (-3.4 to -1.2)	< 0.001	-3.5 (-5.1 to -2.0)	< 0.001	-6.5 (-9.0 to -4.0)	< 0.001
IgA RF (> 25 U/ml)	-0.7 (-1.9 to 0.5)	0.26	-2.6 (-4.3 to -1.0)	0.002	-5.0 (-7.6 to -2.3)	< 0.001
IgM RF(> 25 U/ml)	-1.2 (-2.3 to -0.1)	0.04	-2.8 (-4.4 to -1.3)	< 0.001	-5.5 (-8.0 to -3.0)	< 0.001
DMARD treatment	-1.6 (-2.6 to -0.5)	0.003	-2.6 (-4.3 to -0.9)	0.004	-5.0 (-7.9 to -2.2)	0.001
Prednisolone use	-1.0 (-2.2 to 0.1)	0.07	-1.9 (-3.5 to -0.4)	0.02	-3.6 (-6.2 to -1.0)	0.008

Results from univariate linear regression analyses with DXR-BMD as dependent variable.

Anti-CCP = antibodies to cyclic citrullinated peptide; CI = confidence interval; CRP = C-reactive protein; DMARD = disease-modifying antirheumatic drugs; DXR-BMD = hand bone mineral density estimated by digital X-ray radiogrammetry; ESR = erythrocyte sedimentation rate; HAQ = health assessment questionnaire; Ig = immunoglobulin; RA = rheumatoid arthritis; RF = rheumatoid arthritis.

In multivariate linear regression models, elevated levels of anti-CCP and ESR were independently associated with one-year loss in DXR-BMD (Table 4). This finding was confirmed in multivariate regression models with two and five-year DXR-BMD change as a dependent variable. In addition, elevated baseline CRP levels and DMARD treatment were independently associated with two-year change in DXR-BMD, but not significantly associated with one and five-year change. Disease duration, HAQ and prednisolone use did not alter or influence any of the multivariate models.

**Table 4:** Independent associations between change in DXR-BMD and baseline serological biomarkers (multivariate linear regression analyses)

	One-year DXR-BMD change		Two-year DXR-BMD change		Five-year DXR-BMD change	
			
	B (95% CI)	*P *value	B (95% CI)	*P *value	B (95% CI)	*P *value
Anti-CCP (> 25 U/ml)	-1.7 (-2.8 to -0.7)	0.002	-2.1 (-3.5 to -0.6)	0.006	-5.5 (-7.8 to -3.1)	< 0.001
ESR (> 20 mm/h)	-2.5 (-3.6 to -1.4)	< 0.001	-3.2 (-4.7 to -1.7)	< 0.001	-5.2 (-7.6 to -2.8)	< 0.001
CRP (> 10 mg/l)			-1.8 (-3.4 to -0.3)	0.02		
DMARD treatment			-1.4 (-2.8 to -0.03)	0.05		
Age (years)	-0.02 (-0.07 to 0.02)	0.3	-0.05 (-0.1 to 0.002)	0.06	-0.08 (-0.2 to 0.01)	0.09
Sex (female)	0.3 (-0.8 to 1.5)	0.6	0.1 (-0.3 to 2.8)	0.1	0.5 (-2.1 to 3.1)	0.5
Constant	0.5 (-1.9 to 3.1)	0.7	1.8 (-1.5 to 5.1)	0.3	1.8 (-3.6 to 7.2)	0.5
Adjusted R^2^	21.1%		29.3%		28.5%	

Final models after multivariate linear regression analyses. Dependent variable: DXR-BMD change (%).

Anti-CCP = antibodies to cyclic citrullinated peptide; CI = confidence interval; CRP = C-reactive protein; DMARD = disease-modifying antirheumatic drugs; DXR-BMD = hand bone mineral density estimated by digital X-ray radiogrammetry; ESR = erythrocyte sedimentation rate.

### Predictors of cortical hand bone loss

Possible predictors of cortical hand bone loss were also examined using univariate logistic regression models with loss in cortical hand bone exceeding LSC as the dependent variable. Elevated baseline levels of anti-CCP and ESR increased the odds of cortical hand bone loss at one, two and five-year follow-up in univariate logistic regression analyses (Table 5). Further, increased odds were observed in high levels of IgA RF for one and two years of bone loss and elevated IgM and CRP levels for two- and five-year bone loss. In addition, patients with increased age and HAQ, and DMARD and prednisolone treatment had higher odds of bone loss.

**Table 5:** Cortical hand bone loss and baseline serological biomarkers (univariate logistic regression analyses)

	One-year cortical hand bone loss		Two-year cortical hand bone loss		Five-year cortical hand bone loss	
			
	OR (95% CI)	*P *value	OR (95% CI)	*P *value	OR (95% CI)	*P *value
Age (years)	1.0 (1.0 to 1.0)	0.15	1.0 (1.0 to 1.1)	0.02	1.1 (1.0 to 1.1)	0.01
Sex (female)	1.3 (0.6 to 2.7)	0.52	2.0 (0.9 to 4.5)	0.09	1.0 (0.3 to 3.5)	0.96
Disease duration (years)	1.0 (0.7 to 1.3)	0.85	1.1 (0.8 to 1.5)	0.69	1.1 (0.7 to 1.7)	0.66
HAQ	1.9 (1.1 to 3.4)	0.03	2.5 (1.3 to 5.0)	0.01	1.5 (0.6 to 3.7)	0.41
ESR (> 20 mm/h)	5.2 (2.4 to 11.1)	< 0.001	5.5 (2.2 to 13.7)	< 0.001	4.6 (1.2 to 17.0)	0.02
CRP (> 10 mg/l)	1.9 (0.9 to 4.1)	0.11	4.1 (1.3 to 12.3)	0.01	6.0 (0.8 to 47.5)	0.09
Anti-CCP (> 25 U/ml)	2.7 (1.4 to 5.5)	0.004	3.2 (1.5 to 7.0)	0.003	5.1 (1.5 to 17.0)	0.01
IgA RF (> 25 U/ml)	2.6 (1.2 to 5.7)	0.01	3.3 (1.3 to 8.5)	0.01	2.2 (0.6 to 8.3)	0.23
IgM RF (> 25 U/ml)	1.7 (0.8 to 3.3)	0.14	3.4 (1.5 to 7.8)	0.005	3.8 (1.0 to 14.2)	0.05
DMARD use	2.1 (1.0 to 4.3)	0.05	2.9 (1.3 to 6.3)	0.009	2.5 (0.8 to 7.4)	0.10
Prednisolone use	2.3 (1.1 to 4.6)	0.02	3.5 (1.5 to 8.1)	0.004	4.8 (1.2 to 17.8)	0.02

Results from univariate logistic regression analyses. Dependent variable: cortical hand bone loss exceeding the least significant change (least significant change = -0.79%).

Anti-CCP = antibodies to cyclic citrullinated peptide; CI = confidence interval; CRP = C-reactive protein; DMARD = disease-modifying antirheumatic drugs; ESR = erythrocyte sedimentation rate; HAQ = health assessment questionnaire; Ig = immunoglobulin; OR = odds ratio; RF = rheumatoid arthritis.

Anti-CCP was a consistent and independent predictor of cortical hand bone loss during the five-year follow-up period in multivariate logistic regression analyses (Table 6). Elevated baseline ESR was independently predictive of one- and two-year cortical hand bone loss. Two-year cortical hand bone loss was also predicted by prednisolone use.

**Table 6:** Independent predictors of cortical hand bone loss, results from multivariate logistic regression analyses

	One-year cortical hand bone loss		Two-year cortical hand bone loss		Five-year cortical hand bone loss	
			
	OR (95% CI)	*P *value	OR (95% CI)	*P *value	OR (95% CI)	*P *value
Anti-CCP (> 25 U/ml)	2.2 (1.0 to 4.5)	0.04	2.6 (1.1 to 6.2)	0.03	4.9 (1.4 to 16.7)	0.01
ESR (> 20 mm/h)	4.5 (2.0 to 9.9)	< 0.001	3.5 (1.3 to 9.3)	0.01		
Prednisolone use			4.7 (1.6 to 14.1)	0.006		
Age (years)	1.0 (0.9 to 1.0)	0.62	1.0 (0.9 to 1.1)	0.23	1.1 (1.0 to 1.1)	0.89
Sex (female)	1.0 (0.4 to 2.4)	0.95	0.5 (0.2 to 1.3)	0.14	0.9 (0.2 to 3.4)	0.02
Constant	0.5	0.35	0.4	0.37	0.3	0.31

Final models after multivariate logistic regression analyses. Dependent variable: cortical hand bone loss (yes/no). Anti-CCP = antibodies to cyclic citrullinated peptide; CI = confidence interval; ESR = erythrocyte sedimentation rate; OR = odds ratio.

## Discussion

The main finding in this five-year longitudinal study of patients with RA of short disease duration was that cortical hand bone loss can be independently predicted by elevated levels of anti-CCP and ESR. This finding adds to the understanding of anti-CCP and ESR as important predictors of bone involvement in RA.

The bone involvement in RA has been shown to start in the inflamed synovium that express receptor activator of nuclear factor-κB ligand (RANKL), a cytokine known to mediate osteoclast differentiation and activation [1,2]. Expressed in increased amounts and up-regulated by cytokines such as IL1 and TNFα, RANKL causes the osteoclast to outperform the osteoblast thus causing increased bone resorption and focal bone loss. This focal bone loss in RA is seen as a reduction in trabecular as well as cortical BMD [4,17-19]. In order to target therapy, prognostic factors of this focal damage should be identified. Erosive disease seen in radiographs has across several studies been shown to be predicted by anti-CCP, RF, ESR and CRP [13]. Change in DXA-BMD hand has been found to be inversely correlated to CRP and RF [18,20]. CRP has also been found to be associated with one-year hand DXR-BMD change in the BeST study [21]. In this study we confirm that RF and CRP are associated with DXR-BMD. However, in addition we show that elevated levels of anti-CCP and ESR are independent predictors of DXR-BMD loss. These common predictors support that erosions and focal bone loss have a common cellular mechanism.

Use of corticosteroids in high dosages indisputably causes a wide range of adverse events, including corticosteroid-induced osteoporosis [22]. Results from studies investigating the effect of chronic low-dose glucocorticoid use on bone in RA are conflicting. Although some studies show increased BMD while using low-dose prednisolone, others show bone loss [22,23]. In this study the patients using prednisolone took a daily mean (standard deviation) dosage of 6.5 (2.7) mg, they were older and had higher HAQ scores than those who did not (data not shown). With respect to DMARDs influence on BMD, Schorn and Mowat have demonstrated an increased cortical thickness in RA patients treated with penicillamine [24]. Kalla and colleagues have also shown that DMARD treatment increase the cortical bone mass in RA patients [25]. The DMARD-treated patients in this study had significantly higher anti-CCP levels than the patients not treated with DMARDs, indicating a more severe disease. We found that both DMARDs and prednisolone use was associated with DXR-BMD loss. RA patients with severe disease are prone to experience bone loss due to inflammation and immobility, but they are also more likely to be treated with DMARDs or prednisolone. Therefore, confounding by indication might explain the associations between DMARD and prednisolone treatment and DXR-BMD loss [26]. We also found age and HAQ to be associated with increased bone loss. Increased age and impaired physical function has previously been shown to explain decreased BMD and might thereby interfere with the results [27].

A weakness of this study is a lack of available data on important factors that influence the BMD. There were no available data on use of vitamin D, calcium supplements, hormone replacement therapy or anti-resorptive treatment. Further, there were no available specifications on the different DMARDs used during the five-year period. The menopausal status of the patients was not known. The DXR-BMD loss was larger in women under 50 years than in those older than 50 years. This might be explained by a rapid bone loss in the immediate years following menopause. However, this finding was similar for men, suggesting that menopause did not influence these results. Another weakness of this study was that DXA-BMD measurements were not performed. The observed cortical bone loss could neither be validated against the measured gold standard DXA-BMD, nor could the observed predictors be validated against trabecular bone loss.

## Conclusions

The results of these analyses imply that a hypothetical 40-year-old female RA patient with elevated levels of ESR and anti-CCP would at one-year follow-up have a predicted DXR-BMD loss of 4.2% and an odds of 6.4 for cortical hand bone loss, compared with a similar patient with normal levels of ESR and anti-CCP (calculated from the multivariate regression models presented in Tables 4 and 6). Thus, our findings support that elevated levels of anti-CCP and ESR are important markers that have potential impact on the disease course and should have impact on considerations about treatment strategies in RA patients. Further, this observation adds support to the hypothesis of similar mechanisms being involved in hand bone loss and erosive disease.

## Abbreviations

Anti-CCP: antibodies to cyclic citrullinated peptide; BMD: bone mineral density; CRP: C-reactive protein; DMARD: disease-modifying antirheumatic drugs; DXR: digital X-ray radiogrammetry; DXR-BMD: hand bone mineral density estimated by digital X-ray radiogrammetry; ELISA: enzyme-linked immunosorbent assay; ESR: erythrocyte sedimentation rate; HAQ: health assessment questionnaire; Ig: immunoglobulin; IL1: interleukin 1; LSC: least significant change; RA: rheumatoid arthritis; RANKL: receptor activator of nuclear factor-kappaB ligand; RF: rheumatoid arthritis; TNFα: tumour necrosis factor α.

## Competing interests

The authors declare that they have no competing interests.

## Authors' contributions

PB performed the statistical analyses and prepared the manuscript. MH contributed in the statistical analyses and substantially contributed to the manuscript. SØ organised the clinical data collection. GH organised the DXR-BMD data collection. SWS organised the data collection. PIG organised the immunoassays. CO organised the analyses of ESR and CRP. All the authors contributed to the manuscript. TKK conceived of the study, and participated in its design and coordination and substantially helped to draft the manuscript. All authors read and approved the final manuscript.
